# The Presence of Diffuse Idiopathic Skeletal Hyperostosis (DISH) among Patients with High Burden of Cardiovascular Risk Factors: A Retrospective Study

**DOI:** 10.1155/2024/8877237

**Published:** 2024-07-23

**Authors:** Nadia Khalaily, Leonid Roshkovan, Amir Bieber, Reuven Mader, Shay Brikman

**Affiliations:** ^1^Internal Medicine C Department, Ha'Emek MC, Afula, Israel; ^2^Department of Radiology, University of Pennsylvania, Philadelphia, PA 19104, USA; ^3^Rheumatic Diseases Unit, Ha'Emek MC, Afula, Israel; ^4^Rappaport Faculty of Medicine, Technion, Haifa, Israel

## Abstract

**Background:**

DISH is a systemic condition characterized by ligamentous ossification of at least four contiguous thoracic vertebrae. Prior studies have shown an association between DISH and cardiovascular morbidity.

**Objective:**

To investigate the association between DISH, cardiovascular risk factors, and MACE (myocardial infarction, ischemic stroke, and CV mortality) in patients who underwent coronary angiography between 5/2014 and 4/2015 in Ha'Emek Medical Center through 7 years of retrospective follow-up.

**Methods:**

Two cohorts were studied retrospectively and were defined according to the status of the coronary artery as diagnosed in angiography at enrolment (obstructive vs. nonobstructive coronary heart disease). For the retrospective analysis, we added the status of DISH (according to Resnick's criteria) and defined four cohorts as follows: CHD and DISH (group 1), CHD and no DISH (group 2), no CHD and DISH (group 3), and no CHD and no DISH (group 4). The four groups were followed up retrospectively for a median period of 7 years. Association between DISH and cardiovascular outcomes was studied.

**Results:**

198 patients were included in the study. 100 of them had CHD, and 98 were without significant CHD. At enrolment, DISH was present in 44 patients of CHD group and in 35 of non-CHD (*p* = 0.28 age and sex adjusted). Through the follow-up period, the presence of DISH was not found to be associated with death from any cause, cardiovascular death, ischemic stroke, and MACE. Within the group of non-CHD, there were two additional MI events in DISH (group 3) which was found to be statistically significant.

**Conclusion:**

Among patients with high burden of cardiovascular risk factors undergoing coronary angiography, the presence of DISH was not associated with an increased incidence of MACE.

## 1. Introduction

Diffuse idiopathic skeletal hyperostosis (DISH) is a musculoskeletal condition with a vague pathophysiology, which is characterized by progressive calcification and ossification of ligaments and entheses, according to the radiological criteria of Resnick and Niwayama [1].

At least four contiguous thoracic vertebrae, generally with preserved disk space and absence of sacroiliac inflammation should be present in a thoracic X-ray in order to define DISH. Predilection to the anterior longitudinal ligament of the spine is another important radiological feature yet not mandatory for classification.

The cervical, lumbar spinal segments, peripheral joints, and entheses might also be affected [2].

DISH is usually asymptomatic; however, back pain and reduced range of motion were already reported. Patients affected are often males and elderly with a prevalence of up to 42% for patients over the age of 65 [3].

According to literature, DISH is associated with several cardiovascular risk factors such as diabetes mellitus and metabolic syndrome [4, 5].

Previous retrospective studies investigated the relationship between DISH and cardiovascular events. For instance, Glick et al. showed that Framingham score underestimated the real risk for developing CVD in patients with DISH, especially MI (39% of the patients with DISH developed CVD, while 28.6% were expected to be affected with CVD by the Framingham score at 10 years of follow-up) [6]. According to Zincarelli et al., which showed that DISH prevalence was 30.3% in patients with severe atherosclerotic cardiovascular diseases [7], it should be mentioned that this study was carried out on patients with various cardiovascular diseases.

In addition, a recent published prospective cohort study which included 4624 patients with a median follow-up of 8.7 years showed that DISH is associated with an increased cumulative incidence of ischemic stroke, but not with MACE, myocardial infarction, vascular death, or all-cause mortality [8].

On the other hand, Dan Lantsman et al. found no independent association between DISH and coronary artery calcification score (CACS) after multivariate analysis in 268 individuals with symptomatic chest pain who has undergone coronary estimation with cardiac CT [9].

In light of the confounding results prementioned before, the aim of the present study was to further investigate the association between DISH and cardiovascular events, i.e., ischemic stroke, new myocardial infarction, CV mortality, and MACE, in patients who underwent coronary angiography for the first time in 2014-2015 in Ha'Emek Medical Center, through 7 years of follow-up.

## 2. Materials and Methods

We assumed that the prevalence of DISH in patients with significant coronary artery disease (CAD) will be similar to that in patients undergoing coronary artery bypass surgeries, i.e., 25%. In order to show a difference of 15% in the prevalence of DISH between patients with obstructive CAD compared with patients with nonobstructive CAD, with a power of 80% and alpha of 5% (two tailed), a sample size of 200 patients was established (1 : 1 ratio with 100 patients in each group).

We retrospectively identified patients (aged ≥ 50 y) who underwent percutaneous coronary angiography between 1^st^ May 2014 and 31^th^ April 2015 for various indications (management of chest pain with angina features, coronary evaluation prior to valve replacement, or in patients with suspected ischemia according to noninvasive modalities), and for whom posterior anterior and lateral chest X-ray, thoracic spine, or chest CT scan are available within 12 months of the catheterization.

Patients were excluded if they were younger than 50 years old or if they lacked a chest radiograph or chest tomography performed within one year prior to cardiac catheterization.

Coronary artery tree was assessed invasively by cardiology specialists. Obstructive coronary heart disease was defined as ≥50% stenosis in epicardial vessels above 2 mm in diameter and nonobstructive coronary heart disease as ≤50% obstruction in epicardial vessels above 2 mm in diameter.

We also reviewed the medical records for all eligible adults and collected demographic data (age, sex), cardiovascular risk factors (smoking, hypertension, hyperlipidemia, diabetes mellitus, and hyperuricemia), relevant medical history, concomitant medications, and cardiovascular outcome (ischemic stroke, major adverse cardiovascular events (MACE), myocardial infarction, and total and cardiovascular mortality).

MACE was defined as the composite of fatal myocardial infarction, myocardial infarction, and ischemic stroke.

Chest radiographs were examined by independent radiologist blinded to the metabolic baseline and cardiovascular events, using the Resnick criteria. Scoring was done at enrolment and at the latest image available through the period of follow-up.

Based on these data, we defined 4 groups in our cohort study according to the epicardial coronary status (obstructive vs. nonobstructive as defined above) at enrolment and to DISH status also at enrolment: CHD and DISH (group 1), CHD and no DISH (group 2), no CHD and DISH (group 3), and no CHD and no DISH (group 4).

Our primary outcome was the development of cardiovascular events through the period of follow-up in the 4 groups (stroke, MACE, CV mortality, MI, and death from any cause).

Secondary outcomes were defined as the development of DISH through the period of follow-up and its impact on developing obstructive coronary heart disease.

### 2.1. Statistical Methods

Data and outcomes were extracted from the medical records of the patients retrospectively by N.K; demographic and baseline characteristics of the two groups (CHD vs. non-CHD) were compared by *χ*^2^ or Fisher's exact test in the case of rare occurrence for categorical variables and by *t*-test or Mann–Whitney test in the case of non-normally distributed data for continuous variables. Logistic regression analysis was used to adjust for age and sex. Demographic and baseline characteristics of the 4 groups were compared by *χ*^2^ or Fisher's exact test in the case of rare occurrence for categorical variables and by one-way ANOVA or the Kruskal-Wallis test in the case of non-normally distributed data for continuous variables. Multinomial logistic regression analysis was used to adjust for age and sex. The Kaplan-Meier survival analysis was performed to evaluate the development of cardiovascular events. The Cox regression analysis was performed to adjust for age, sex, smoking, hypertension, diabetes, hyperlipidemia, and hyperuricemia. Similar analyses were performed to evaluate the development of cardiovascular events between the DISH and never DISH groups. Statistical significance was considered to be *p* < 0.05. All analyses were performed by IBM SPSS version 24 (IBM Corp, Armonk, N.Y.).

### 2.2. Ethics

The study was approved by the local institutions' ethics committee (Helsinki subcommittee). Due to the retrospective nature of the study, patient consent was waived by the ethics committee.

## 3. Results

### 3.1. Baseline Characteristics

**Figure 1:**
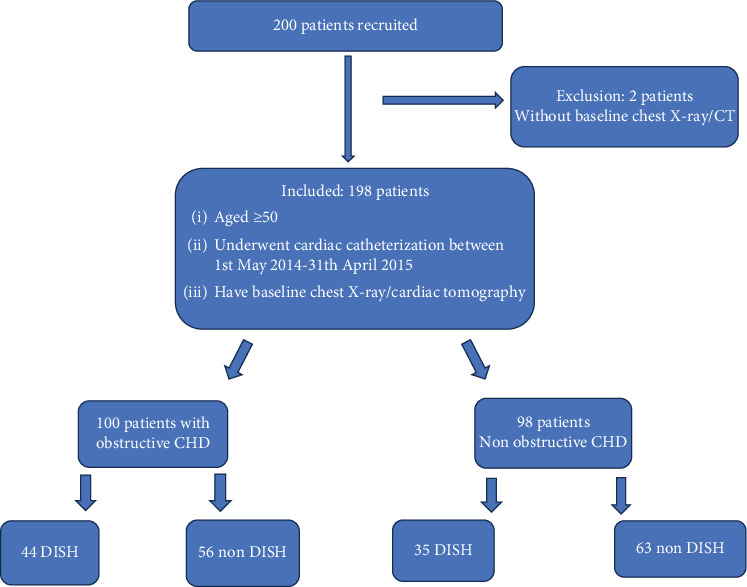
Flow chart of the study design.

Figure 1 presents flow diagram for our retrospective study; 200 patients were recruited, 198 patients were eligible and included in our retrospective cohort study, and two patients have no follow-up medical record available, hence censored from the analysis (118 males; mean age 64.4 years, range 49.3-89.6). Of the 198 patients, 100 (50.5%) had obstructive coronary heart disease and 79 (39.9%) had DISH (44 of them had CHD, 35 non-CHD, *p* = 0.28).

Table 1 presents baseline patient characteristics by coronary status at recruitment. DISH at enrolment was not associated with coronary status (OR: 1.41; 95% CI: 0.80-2.50).

Patients with DISH were significantly older than those who did not have (*p* < 0.006). After adjusting for age and sex, patients who developed DISH tended to have higher rate of diabetes (72.7 vs. 43.8%, *p* < 0.08) as compared to patients who did not have DISH (Table S1 describing baseline patient characteristics by group: patients diagnosed with DISH at enrolment, patients who developed DISH, and those who did not develop DISH—in the supplementary Appendix, available with the full text of this article). Also, see supplementary table 3 for details about patient characteristics by coronary status at baseline and diffuse idiopathic skeletal hyperostosis at the end of the follow-up period.

DISH in our study was more prevalent in older patients; Figure S1 (in the supplementary Appendix) presents the distribution of DISH across age groups at enrolment (*χ*^2^ = 16.60, *p* = 0.018) which was statistically significant.

Patients were followed a median of 76.5 months (median: 87, range 0-92 months). There was no statistically significant difference in the length of time followed between the groups (Kruskal‐Wallis = 0.25, *p* = 0.882). 12 patients lost to follow-up.

In our retrospective analysis, we divided patients into 4 groups according to the coronary status and DISH status at enrolment, as prementioned above CHD and DISH (group 1), CHD and no DISH (group 2), non-CHD and DISH (group 3), and non-CHD and no DISH (group 4), 22.2%, 28.3%, 17.7%, and 31.8%, accordingly.

Table 2 presents patient characteristics by coronary status and DISH status as presented at baseline. The 4 groups differed in age (*F*(3,194) = 3.79, *p* = 0.01), sex (*χ*^2^(3) = 13.36, *p* = 0.004), smoking habit (*χ*^2^(6) = 13.37, *p* = 0.038), and rates of hyperlipidemia (*χ*^2^(3) = 8.41, *p* = 0.038).

### 3.2. Cardiovascular Outcomes during Follow-Up

Through the follow-up period, we assessed the DISH status according to the last updated imaging and development of cardiovascular outcomes: death from any cause, cardiovascular death, ischemic stroke, MACE, and MI.

During follow-up, there were 43 deaths from any cause and 7 cardiovascular deaths. The Kaplan-Meir survival analysis revealed no statistically significant difference in months survived between DISH and never DISH groups for deaths from any cause (Mantel‐Cox = 0.002, *p* = 0.96; mean DISH survival time: 77.8 months vs. never DISH: 81.8 months), cardiovascular deaths (Mantel − Cox = 0.31, *p* = 0.579; mean DISH survival time: 88.4 months vs. never DISH: 90.3 months), and strokes (Mantel − Cox = 2.70, *p* = 0.10; mean DISH: 84.5 months vs. never DISH: 89.8 months); even after adjusting for age, sex, smoking, hypertension, diabetes, hyperlipidemia, and hyperuricemia, there remained no statistically significant differences. In addition, there were no differences between DISH and never DISH in new MI (Mantel‐Cox = 0.91, *p* = 0.34), MACE (Mantel‐Cox = 1.20, *p* = 0.27), 2 or more MACE (Mantel − Cox = 1.58, *p* = 0.21), or CHF (Mantel‐Cox = 0.36, *p* = 0.83) (table S2 describing negative cardiovascular events by DISH status at the end of the follow-up period is presented in the supplementary Appendix).

### 3.3. DISH in relation to Cardiovascular Outcomes

During the period of follow-up, 53 patients developed new MACE, 14 patients developed new stroke; there was 45 deaths from any cause (2 of them had no updated imaging and unknown DISH status through the follow-up period), and 8 developed cardiovascular deaths (one of them without updated imaging and unknown status of DISH).

Of the 186 patients with known DISH status at the end of follow-up period, 46 (24.7%) patients had nonobstructive CHD and never had DISH, 41 (22.0%) had no obstructive CHD and DISH, 50 (26.9%) obstructive CHD and no DISH, and 49 (26.3%) obstructive CHD and DISH. Table S3 (in the supplementary Appendix) presents patient characteristics by coronary status at baseline and DISH status occurring through the follow-up period, and Table 3 presents cardiovascular events by DISH status at the end of the follow-up period.

The four groups differed in age, sex, and rate of hyperlipidemia and smoking. There was no statistically significant difference in age among patients who had nonobstructive CHD. The four groups had statistically significant different distribution of males (*χ*^2^ = 13.11, *p* = 0.004), smoking habits (*χ*^2^ = 15.26, *p* = 0.018), and rates of hyperlipidemia (*χ*^2^ = 7.70, *p* = 0.051).  Table 4 presents cardiovascular events by group (patients with CHD and no CHD with DISH and no DISH).

Planned post hoc test revealed that there were no statistically significant differences between nonobstructive CHD DISH and no DISH patients except for the rate of hypertension which was higher in patients with DISH (92.7 vs. 71.7%, *χ*^2^ = 6.34, *p* = 0.012). There remained a significant difference in the rate of hypertension after correcting for age and sex (*χ*^2^ = 5.02, *p* = 0.025).

Among patients with obstructive CHD, there were no statistically significant differences between DISH and no DISH patients except age (mean difference 5.9 years, *t* = 2.86, *p* = 0.005; at follow-up: mean difference 5.7 years, *t* = 3.01, *p* = 0.003) and smoking habit (*χ*^2^ = 10.56, *p* = 0.005). After correcting for age and sex, smoking habit was no longer statistically significant (*χ*^2^ = 3.72, *p* = 0.156).

The study analyzed four groups based on coronary status and DISH at enrollment. There was no significant difference in deaths from any cause, cardiovascular deaths, or strokes. After adjusting for cofounders such as age, sex, obesity, smoking, hypertension, diabetes, and hyperlipidemia, there was still no significant differences in deaths from any cause (*χ*^2^ = 0.12, *p* = 0.989), cardiovascular deaths (*χ*^2^ = 3.56, *p* = 0.313), or strokes (*χ*^2^ = 1.72, *p* = 0.633).

**Figure 2:**
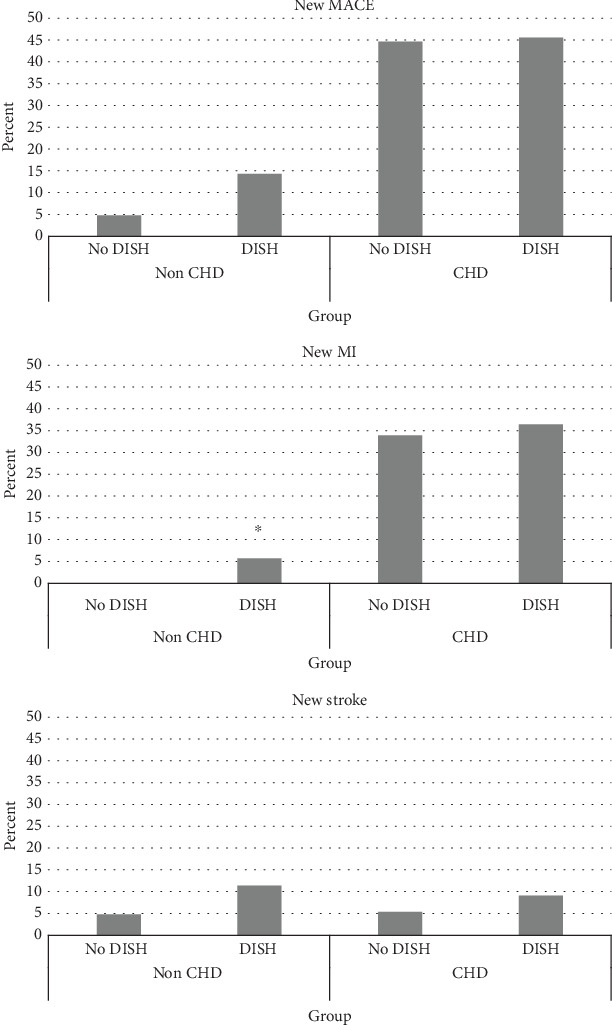
Adverse cardiovascular outcomes by group. ⁣^∗^DISH vs no DISH *p* < 0.05.

**Figure 3:**
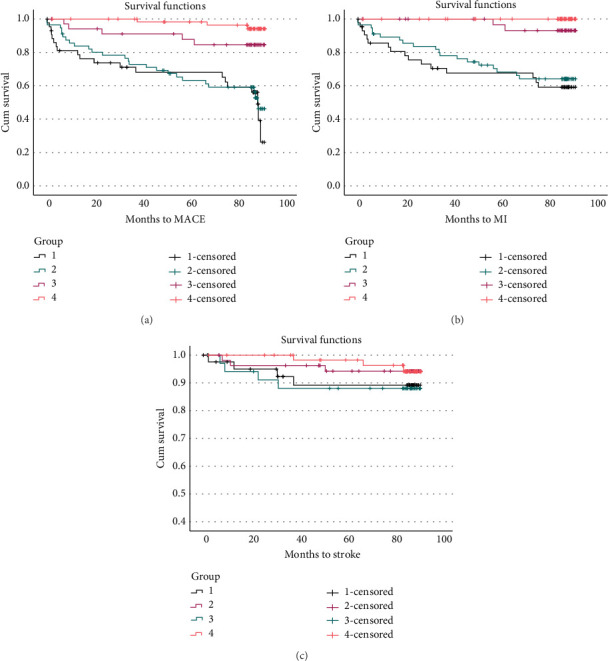
Survival function of cardiovascular outcomes. Survival analysis (KM) revealed a statistically significant difference in months survived between the 4 groups in MACE (Mantel − Cox = 18.17, *p* < 0.001) and new MI (Mantel − Cox = 38.12, *p* < 0.001), yet there was no statistically significant difference between the 4 groups in strokes (Mantel − Cox = 2.40, *p* = 0.494). (a–c) Group 1 CHD no DISH, Group 2 CHD and DISH, Group 3 no CHD DISH, and Group 4 no CHD no DISH. Abbreviations: CHD: coronary heart disease; DISH: diffuse idiopathic skeletal hyperostosis.

Survival analysis revealed a statistically significant difference between the four groups in MACE (Mantel − Cox = 18.17, *p* < 0.001) and MI (Mantel − Cox = 38.12, *p* < 0.001) (Figure 2). Stratification by presence or absence of obstructive coronary heart disease at recruitment revealed no differences in MACE or MI between DISH and no DISH (MACE: Mantel − Cox = 0.214, *p* = 0.644; MI: Mantel − Cox = 0.41, *p* = 0.522); among those who did not have obstructive CHD at recruitment, there was a significant difference in new MI (Mantel − Cox = 3.82, *p* = 0.050) and a borderline difference in MACE (Mantel − Cox = 2.88, *p* = 0.090); the Kaplan-Meir for survival analysis between the four groups is shown in Figure 3.

Through 7 years of follow-up, 11 patients developed DISH; the presence of DISH was not found to be associated with death from any cause, cardiovascular death, ischemic stroke, and MACE in comparison to patients who did not develop DISH. Within the group of non-CHD, there were two additional MI events in DISH (group 3) which was found to be statistically significant.

## 4. Discussion

In our research, we found that DISH did not independently predict cardiovascular events in patients with high burden risk factors, through 7 years of follow-up; the presence of DISH was not found to be associated with death from any cause, cardiovascular death, ischemic stroke, and MACE in comparison to patients who did not develop DISH (table S2); the percentage of the aforementioned cardiovascular outcomes was higher in patients who developed DISH, but these differences were not statistically significant.

Our study has shown that in patients with nonobstructive coronary disease, patients with DISH are more prone to develop MI (2 patients, 5.7%, *p* 0.05), similar to the results of Glick et al. which included 45 patients with DISH and 47 controls without DISH (both groups included patients without known CV diseases) [6]. In this study, the incidence of MI over the 10-year period was higher in the DISH group (24.4% vs. 4.3%, accordingly, *p* 0.0055). Compared to our study, it might be that the 7-year follow-up was too short. In addition, we only had a small sample of patients diagnosed with DISH in the non-CHD group (group 3; 35 patients) making it hard to generalize our conclusions.

We hypothesized that patients with DISH are more prone to develop cardiovascular morbidity and mortality, with a prevalence of at least 10% more than patients not diagnosed with DISH. We did not reach these percentages in our research; it could be explained by the small sample size and relatively short period of follow-up.

In comparison to previous research, the percentage of DISH in our study at enrolment (39.9%) was higher in comparison to Harlianto et al. where the prevalence of DISH was 30.3% and 9.4% [7, 10]. This could be ascribed by the older population of our study (64.4 ± 9.6) and the high burden of cardiovascular risk factors which none of the aforementioned researchers has reached.

It should be noted that the patients in our study had a higher burden of risk factors than expected in the general population (50.8% obese, 81.2% with hypertension, 83.8% with hyperlipidemia, and 49.5% with DM), and 100 of them already had developed obstructive coronary heart disease, so that DISH status could not be considered as risk factor for developing cardiovascular outcomes at this late stage. Also, the population in our study was older with a mean age of 64.4 ± 9.6 (49.3-89.6) with a higher prevalence of DISH in older populations as shown in figure S1, where the cardiovascular risk factors were also higher in these elderly patients; based on these results, it is hard to establish if DISH is a marker of old age or a metabolic marker associated with CV risk factors and outcomes [11]. DISH prevalence in younger ages (as shown in Figure S1 for patients under 60) warrants further investigation. A possible explanation could be the association between established coronary artery disease or high cardiovascular risk factors in this population. Subanalysis studies stratified by age subgroups are necessary to confirm this hypothesis.

The percentage of cardiovascular outcomes (MACE, MI, and ischemic stroke) was higher in patients who had DISH or developed DISH through the period of follow-up in comparison to patients who did not develop (31%, 22.2%, and 10%); none of these differences was statistically significant. Our results are similar to the results of Dan Lantsman et al. which assessed coronary artery disease via two different scoring systems in CT; the score in the DISH group was higher but was not significant; both studies have small study sample and the retrospective manner, and these findings may suggest more complex and noncausal relationship between DISH and coronary disease [12].

Two studies, one by Harlianto et al. and another by Hirota et al., investigated the association between aortic calcifications and DISH. These calcifications are as a result of atherosclerotic mechanisms of the vessel. Both found increased association between aortic calcifications and DISH [13, 14]. Specifically, Hirota et al. were able to show a significant increased prevalence of DISH among patients undergone cardiovascular event vs. no event. Compared to our study, they suggest a stronger correlation between the two conditions, pointing to a shared pathogenic process. In contrast, in our study, we could not prove that DISH was an independent risk factor for cardiovascular event, indicating that other metabolic factors might affect both conditions.

Our study has some limitations. The study population included 198 patients which might be underpowered according to smaller differences in terms of metabolic syndrome and other confounders between the groups. The retrospective nature of our study should be taken into consideration when interpreting confounders, since not all confounders could be normalized. Yet, most important and known metabolic variables were integrated in our model.

## 5. Conclusion

In our study, DISH was not an independent risk factor for cardiovascular events in a high-risk cardiovascular patient group. Further studies are needed to explore the contribution of DISH and its association with other cardiovascular risk determinants on cardiovascular outcomes.

## Figures and Tables

**Table 1:** Baseline patient characteristics by coronary status in angiography at recruitment.

	Total group (*N* = 198)	CHD (*N* = 100)	Non-CHD (*N* = 98)	*p* (age and sex adjusted)
Age (range)	64.4 ± 9.6 (49.3-89.6)	64.9 ± 10.8 (49.3-89.6)	63.9 ± 8.3 (50.5-89.4)	—
Sex (male)	118 (59.6)	72 (72.0)	46 (46.9)	<0.001^1^
Obesity	100 (50.8)	43 (43.0)	57 (58.8)^a^	0.08
DISH	79 (39.9)	44 (44.0)	35 (35.7)	0.28
Smoking				0.36
Past	29 (14.6)	18 (18.0)	11 (11.2)	0.20
Active	53 (23.6)	30 (30.0)	23 (23.5)	0.33
Hyperuricemia	57 (28.8)	23 (23.0)	34 (34.7)	0.16
Gout	6 (3.0)	4 (4.0)	2 (2.0)	0.62
Hypertension	160 (81.2)	82 (82.0)	78 (80.4)	0.38
PVD	18 (9.1)	14 (14.0)	4 (4.1)	0.06
Atrial fibrillation	36 (18.2)	15 (15.0)	21 (21.4)	0.20
Hyperlipidemia	166 (83.8)	91 (91.0)	75 (76.5)	0.004
Diabetes	98 (49.5)	49 (49.0)	49 (50.0)	0.92
Use of				
Beta blockers	123 (64.1)	69 (69.7)	54 (58.1)	0.03
NOAC				0.32
Active	19 (9.8)	6/99 (6.1)	13/94 (13.8)	0.13
Past	8 (4.1)	4/99 (4.0)	4/94 (4.3)	0.95
Aspirin				
Active	115 (59.9)	72 (72.7)	43 (46.2)	<0.001
Past	44 (22.9)	27 (27.3)	17 (18.7)	0.17
Anticoagulants				<0.001
NOAC	31 (16.3)	28 (28.3)	3 (3.3)	
Coumadin	59 (31.1)	55 (55.6)	4 (4.4)	
Calcium channel blockers	59 (31.1)	20 (20.2)	39 (42.9)	<0.001
Metformin	62 (32.6)	31 (31.3)	31 (34.1)	0.87
Insulin	33 (27.5)	20 (20.2)	13 (14.4)	0.14
Other diabetes medication	48 (25.3)	24 (24.2)	24 (26.4)	0.74
Lipid lowering agents	130 (68.4)	77 (77.8)	53 (58.2)	0.008
Allopurinol	7 (3.7)	4 (4.0)	3 (3.3)	0.88
ACEi/ARB	116 (58.8)	64 (64.6)	52 (57.1)	0.21

Abbreviations: CHD: coronary heart disease; DISH: diffuse idiopathic skeletal hyperostosis; PVD: peripheral vascular disease; NOAC: new oral anticoagulation; ACEi: angiotensin-converting enzyme inhibitor; ARB: angiotensin II receptor blocker. ^1^Age adjusted.

**Table 2:** Patients' characteristics by coronary status and diffuse idiopathic skeletal hyperostosis (DISH) status as presented at baseline.

	Group						All 4 groups	Non-CHD vs. CHD	
	Non-CHD at baseline			CHD disease at baseline					
	No DISH (*N* = 63)	DISH (*N* = 35)	*p*	No DISH (*N* = 56)	DISH (*N* = 44)	*p*	*p*	No DISH	DISH
Age	62.4 ± 8.5 (50.5-89.4)	66.6 ± 7.1 (51.8-80.9)	0.01	62.8 ± 10.0 (50.0-89.6)	67.5 ± 11.2 (49.3-88.4)	0.03	0.01	>0.99	>0.99
Age at end of follow-up	69.0 ± 8.0 (57.5-92.6)	73.0 ± 7.4 (58.9-88.5)	0.02	69.4 ± 9.3 (57.4-92.5)	73.7 ± 10.1 (57.7-94.7)	0.007	0.01	0.80	0.34
Follow-up (months)	79.1 ± 20.3 (2-92)	75.6 ± 24.6 (2-92)	0.96	78.6 ± 20.1 (6-91)	70.6 ± 31.9 (0-91)	0.95	0.91	0.50	0.77
Sex (male)	31 (49.2)	15 (42.9)	0.55	41 (73.2)	31 (70.5)	0.76	0.004	0.007	0.01
Obesity	36 (57.1)	21 (61.8)	0.66	21 (37.5)	22 (50.0)	0.21	0.09	0.04	0.30
Smoking			0.50			0.02	0.04	0.07	0.09
Past	9 (14.3)	2 (5.7)		8 (14.3)	10 (22.7)				
Active	14 (22.2)	9 (25.7)		23 (41.1)	7 (15.9)				
Hyperuricemia	24 (38.1)	10 (28.6)	0.34	9 (16.1)	14 (31.8)	0.06	0.06	0.007	0.76
Gout	1 (1.6)	1 (2.9)	>0.99	2 (3.6)	2 (4.5)	>0.99	0.85	0.60	>0.99
Hypertension	45 (72.6)	33 (94.3)	0.01	47 (83.9)	35 (79.5)	0.57	0.06	0.14	0.10
PVD	3 (4.8)	1 (2.9)	>0.99	9 (16.1)	5 (11.4)	0.50	0.09	0.04	0.22
Atrial fibrillation	12 (19.0)	9 (25.7)	0.44	6 (10.7)	9 (20.5)	0.18	0.31	0.21	0.58
Hyperlipidemia	47 (74.6)	28 (80.0)	0.55	50 (89.3)	41 (93.2)	0.73	0.04	0.04	0.10
Diabetes	29 (46.0)	20 (57.1)	0.29	27 (48.2)	22 (50.0)	0.86	0.76	0.81	0.53

Abbreviations: CHD: coronary heart disease; PVD: peripheral vascular disease.

**Table 3:** Cardiovascular events by DISH status at the end of the follow-up period.

	DISH (*N* = 90)	Never DISH (*N* = 96)	*p* (*χ*^2^)	*p* (KM)
MACE (CB)	28 (31.1)	24 (25.0)		0.27
Number of MACE (median; range)	0.54 ± 1.03 (0; 0-5)	0.35 ± 0.71 (0; 0-4)	0.29	
Number of MACE			0.52	
0	62 (68.9)	72 (75.0)		
1	16 (17.8)	16 (16.7)		
2	8 (8.9)	7 (7.3)		
>2	4 (4.4)	1 (1.0)		
MACE ≥ 2	12 (13.3)	8 (8.3)	0.27	0.21
New MI	20 (22.2)	17 (17.7)	0.44	0.34
Stroke	9 (10.0)	4 (4.2)		0.10
Number of strokes			0.13	
0	81 (90.0)	92 (95.8)		
1	7 (7.8)	2 (2.1)		
2	0 (0.0)	1 (1.0)		
>2	2 (2.2)	1 (1.0)		
Stroke ≥ 2	2 (2.2)	2 (2.0)		
CHF	15 (16.7)	20 (20.8)		0.55
Cardiovascular death	4 (4.4)	3 (3.1)		0.58
Death from any cause	20 (22.2)	23 (24.0)		0.96

Abbreviation: DISH: diffuse idiopathic skeletal hyperostosis; MACE: major adverse cardiovascular events; MI: myocardial infarction; CHF: congestive heart failure; KM: Kaplan-Meier.

**Table 4:** Cardiovascular events by group.

	Non-CHD			CHD			All 4
	No DISH (*N* = 63)	DISH (*N* = 35)	*p*	No DISH (*N* = 56)	DISH (*N* = 44)	*p*	*p*
MACE (CB)	3 (4.8)	5 (14.3)	0.09	25 (44.6)	20 (45.5)	0.64	<0.001
Number of MACE (median; range)	0.06 ± 0.30 (0; 0-2)	0.23 ± 0.73 (0; 0-4)	0.10^1^	0.62 ± 0.84 (0; 0-4)	0.84 ± 1.21 (0; 0-5)	0.63^1^	<0.001
Number of MACE			0.11^2^			0.52^2^	<0.001^2^
0	60 (95.2)	30 (85.7)		31 (55.4)	24 (54.5)		
1	2 (3.2)	4 (11.4)		17 (30.4)	10 (22.7)		
2	1 (1.6)	0 (0.0)		7 (12.5)	7 (15.9)		
>2	0 (0.0)	1 (2.9)		1 (1.8)	3 (6.8)		
MACE ≥ 2	*1 (1.6*)	*1 (2.9*)	0.64	*8 (14.3*)	*10 (22.7)*	0.17	<0.001
New MI	0 (0.0)	2 (5.7)	0.05	19 (33.9)	16 (36.4)	0.52	<0.001
Stroke	3 (4.8)	4 (11.4)	0.20	3 (5.4)	4 (9.1)	0.37	0.49
Number of strokes			0.35^2^			0.82^2^	0.57^2^
0	60 (95.2)	31 (88.6)		53 (94.6)	40 (90.9)		
1	2 (3.2)	3 (8.6)		2 (3.6)	3 (6.8)		
2	1 (1.6)	0 (0.0)		0 (0.0)	0 (0.0)		
>2	0 (0.0)	1 (2.9)		1 (1.8)	1 (2.3)		
Stroke ≥ 2	1 (1.6)	1 (2.9)		1 (1.8)	1 (2.3)		
CHF	16 (25.4)	7 (20.0)	0.60	8 (14.3)	6 (13.6)	0.95	0.40
Cardiovascular death	2 (3.2)	1 (2.9)	0.95	2 (3.6)	3 (6.8)	0.40	0.70
Death from any cause	11 (17.5)	9 (25.7)	0.33	14 (25.0)	11 (25.0)	0.78	0.66

Abbreviations: CHD: coronary heart disease; DISH: diffuse idiopathic skeletal hyperostosis; MACE: major adverse cardiovascular events; MI: myocardial infarction; CHF: congestive heart failure. Values in italic refers to a possible association between DISH and increased adverse events in patients with obstructive coronary disease. ^1^Kruskal-Wallis test. ^2^*χ*^2^. *p* values are from the Mantel-Cox log rank test unless otherwise noted.

## Data Availability

The data is withheld at the investigator's dataset and is not freely available. Encoded data can be requested and will need an extra ethical approval.

## Abbreviations

CHD: Coronary heart disease
DISH: Diffuse idiopathic skeletal hyperostosis
PVD: Peripheral vascular disease
CV: Cardiovascular
DM: Diabetes mellitus
MI: Myocardial infarction
CVD: Cardiovascular disease
CHF: Congestive heart failure
CABG: Coronary artery bypass grafting
HVR: Heart valve replacement.

## Additional Points

*Key Point*. The presence of DISH was not associated with an increased incidence of MACE among patients with burden of cardiovascular.

## Supplementary Materials

Supplementary Materials Additional supporting information can be found online in the Supporting Supplementary Appendix is available in the attached file, with the full text of this article: Table S1: baseline patient characteristics by group and multinomial logistic regression analysis of factors associated with diffuse idiopathic skeletal hyperostosis (DISH). Table S2: negative cardiovascular events by DISH status at the end of the follow-up period. Table S3: patient characteristics by coronary status at baseline and diffuse idiopathic skeletal hyperostosis at the end of the follow-up period. Figure S1: prevalence of DISH at enrolment according to age.

## References

[1] Resnick D., Niwayama G. (1976). Radiographic and pathologic features of spinal involvement in diffuse idiopathic skeletal hyperostosis (DISH). *Radiology*.

[2] Mader R., Sarzi-Puttini P., Atzeni F. (2009). Extraspinal manifestations of diffuse idiopathic skeletal hyperostosis. *Rheumatology*.

[3] Holton K. F., Denard P. J., Yoo J. U. (2011). Diffuse idiopathic skeletal hyperostosis and its relation to back pain among older men: the MrOS study. *Seminars in Arthritis and Rheumatism*.

[4] Mader R., Novofestovski I., Adawi M., Lavi I. (2009). Metabolic syndrome and cardiovascular risk in patients with diffuse idiopathic skeletal hyperostosis. *Seminars in Arthritis and Rheumatism*.

[5] Okada E., Ishihara S., Azuma K. (2021). Metabolic syndrome is a predisposing factor for diffuse idiopathic skeletal hyperostosis. *Neurospine*.

[6] Glick K., Novofastovski I., Schwartz N., Mader R. (2020). Cardiovascular disease in diffuse idiopathic skeletal hyperostosis (DISH): from theory to reality - a 10-year follow-up study. *Arthritis Research & Therapy*.

[7] Zincarelli C., Iervolino S., di Minno M. N. D. (2012). Diffuse idiopathic skeletal hyperostosis prevalence in subjects with severe atherosclerotic cardiovascular diseases. *Arthritis Care & Research*.

[8] Harlianto N. I., Oosterhof N., Foppen W. (2022). Diffuse idiopathic skeletal hyperostosis is associated with incident stroke in patients with increased cardiovascular risk. *Rheumatology*.

[9] Dan Lantsman C., Brodov Y., Matetzky S. (2023). No correlation between diffuse idiopathic skeletal hyperostosis and coronary artery disease on computed tomography using two different scoring systems. *Acta Radiologica*.

[10] Harlianto N. I., Westerink J., Hol M. E. (2022). Patients with diffuse idiopathic skeletal hyperostosis have an increased burden of thoracic aortic calcifications. *Rheumatology Advances in Practice*.

[11] Mader R., Lavi I. (2009). Diabetes mellitus and hypertension as risk factors for early diffuse idiopathic skeletal hyperostosis (DISH). *Osteoarthritis and Cartilage*.

[12] Dan Lantsman C., Herman A., Verlaan J. J., Stern M., Mader R., Eshed I. (2018). Abdominal fat distribution in diffuse idiopathic skeletal hyperostosis and ankylosing spondylitis patients compared to controls. *Clinical Radiology*.

[13] Harlianto N. I., Westerink J., Hol M. E. (2022). Patients with diffuse idiopathic skeletal hyperostosis have an increased burden of thoracic aortic calcifications. *Rheumatology Advances in Practice*.

[14] Hirota R., Teramoto A., Yoshimoto M. (2023). Osteophyte bridge formation correlates with vascular calcification and cardiovascular disease in diffuse idiopathic skeletal hyperostosis. *Journal of Clinical Medicine*.

