# Supplementary material for: The Presence of Diffuse Idiopathic Skeletal Hyperostosis (DISH) among Patients with High Burden of Cardiovascular Risk Factors: A Retrospective Study
Source: Int J Rheumatol. 2024 Jul 23;2024:8877237. doi: 10.1155/2024/8877237 (PMC11949591; doi:10.1155/2024/8877237)
Supplement: Supplementary Materials — Additional supporting information can be found online in the Supporting Supplementary Appendix is available in the attached file, with the full text of this article: Table S1: baseline patient characteristics by group and multinomial logistic regression analysis of factors associated with diffuse idiopathic skeletal hyperostosis (DISH). Table S2: negative cardiovascular events by DISH status at the end of the follow-up period. Table S3: patient characteristics by coronary status at baseline and diffuse idiopathic skeletal hyperostosis at the end of the follow-up period. Figure S1: prevalence of DISH at enrolment according to age. [file 8877237.f1.docx]

Appendix

Table S1 presents the Baseline patient characteristics by group (patients diagnosed with DISH at enrolment, patients who developed DISH through the period of follow up, and patients who didn’t develop DISH through period of follow up) and multinomial logistic regression analysis of factors associated with diffuse idiopathic skeletal hyperostosis

Table S1. Baseline patient characteristics by group and multinomial logistic regression analysis of factors associated with diffuse idiopathic skeletal hyperostosis (DISH)

|  |  | Group |  |  | Age and Sex adjusted OR (95% CI) | | Age and Sex adjusted |
| --- | --- | --- | --- | --- | --- | --- | --- |
|  | Got DISH (N=11) | DISH (at  enrollment) (N=79) | Never DISH (N=96) | unadj p | DISH vs. Never DISH | Got DISH vs. Never DISH | p |
| Age | 64.9±10.0  (50.5-77.4) | 67.1±9.6  (49.3-88.4) | 62.5±9.2  (50.0-89.6) | .006 | 1.05 (1.02-1.09) | 1.03 (0.96-1.10) | .0061 |
| Age at end of follow up | 72.2±10.0  (57.5-84.6) | 73.4±9.0  (57.7-94.7) | 69.1±8.4  (57.4-92.6) | .006 | -------- | ---------- |  |
| Time followed (months) | 87.2±2.2  (66.8, 84-92) | 72.8±28.7  (66.1, 0-  92) | 78.5±19.3  (60.1, 6-92) | .88 | --- | --- |  |
| Sex (male) | 6 (54.5) | 46 (58.2) | 59 (61.5) | .86 | 1.00 (0.53-1.88) | 0.82 (0.23-2.90) | .952 |
| Obesity | 4 (36.4) | 43 (55.1) | 47 (49.0) | .44 | 1.55 (0.82-2.93) | 0.63 (0.17-2.39) | .23 |
| **Smoking**  Past Active | 2 (18.2)  1 ( 9.1) | 12 (15.2)  16 (20.3) | 15 (15.6)  33 (34.4) | .14  .97  .04 | 0.87 (0.35-2.14)  0.59 (0.27-1.29) | 0.83 (0.16-4.46)  0.20 (0.02-1.71) | .40 |
| Hyperuricemia | 3 (27.3) | 24 (30.4) | 24 (25.0) | .75 | 0.92 (0.44-1.92) | 0.88 (0.19-4.07) | .99 |
| Gout | 0 (0.0) | 3 (3.8) | 3 (3.1) | >.99 | 1.23 (0.23-6.50) | 0.00 (0.00- | .68 |
| Hypertension | 10 (90.9) | 68 (86.1) | 75 (78.1) | .38 | 1.62 (0.70-3.74) | 2.60 (0.31-21.85) | .40 |
| PVD | 1 (9.1) | 6 (7.6) | 11 (11.5) | .72 | 0.52 (0.17-1.54) | 0.72 (0.08-6.90) | .49 |
| Atrial fibrillation | 1 (9.1) | 18 (22.8) | 16 (16.7) | .48 | 1.07 (0.48-2.40) | 0.33 (0.03-3.17) | .59 |
| Hyperlipidemia | 10 (90.9) | 69 (87.3) | 80 (83.3) | .74 | 1.42 (0.59-3.45) | 2.10 (0.25-17.91) | .67 |
| Diabetes | 8 (72.7) | 42 (53.2) | 42 (43.8) | .13 | 1.65 (0.88-3.08) | 3.57 (0.88-14.44) P<.08 | .095 |

Abbreviations: DISH, diffuse idiopathic skeletal hyperostosis; PVD, peripheral vascular disease. unadj=unadjusted

^1^adjusted for sex ^2^ adjusted for age

Table S2: Negative cardiovascular events by DISH status at the end of the follow up period

the presence of DISH was not found to be associated with death from any cause, cardiovascular death, ischemic stroke and MACE in comparison to patients who didn’t develop DISH

|  | DISH (N=90) | Never DISH (N=96) | P (χ^2^) | p^1^(KM) |
| --- | --- | --- | --- | --- |
| MACE (CB) | 28 (31.1) | 24 (25.0) |  | .27 |
| Number of MACE (Median; range) | 0.54±1.03  (0; 0-5) | 0.35±0.71  (0; 0-4) | .29 |  |
| **Number of MACE**  0  1  2  >2 | 62 (68.9)  16 (17.8)  8 (8.9)  4 (4.4) | 72 (75.0)  16 (16.7)  7 (7.3)  1 (1.0) | .52 |  |
| ***MACE ≥2*** | ***12 (13.3***) | ***8 (8.3***) | .27 | .21 |
| New MI | 20 (22.2) | 17 (17.7) | .44 | .34 |
| Stroke | 9 (10.0) | 4 (4.2) |  | .10 |
| Number of strokes 0  1  2  >2 | 81 (90.0)  7 (7.8)  0 (0.0)  2 (2.2) | 92 (95.8)  2 (2.1)  1 (1.0)  1 (1.0) | .13 |  |
| Stroke***≥2*** | 2 (2.2) | 2 (2.0) |  |  |
| CHF | 15 (16.7) | 20 (20.8) |  | .55 |
| Cardiovascular death | 4 (4.4) | 3 (3.1) |  | .58 |
| Death from any cause | 20 (22.2) | 23 (24.0) |  | .96 |

Abbreviation: DISH, diffuse idiopathic skeletal hyperostosis; MACE, major adverse cardiovascular events; MI, myocardial infarction; CHF, congestive heart failure; KM, Kaplan Meier.

Table S3: Patient characteristics by coronary status at baseline and diffuse idiopathic skeletal hyperostosis at the end of follow up period.

|  | Group | | | | | |  | | |
| --- | --- | --- | --- | --- | --- | --- | --- | --- | --- |
|  | non CHD at Baseline | | | CHD at Baseline | | | All 4 groups | non CHD vs.  CHD | |
|  | Never DISH (N=46) | DISH (N=41) | p | Never DISH (N=50) | DISH (N=49) | p | p | Never DISH | DISH |
| Age | 62.9±8.5 | 65.4±7.6 | .16 | 62.1±9.8 | 68.0±10.9 | .005 | .009 | .66 | .20 |
|  | (50.7-89.4) | (50.5-80.9) |  | (50.0-89.6) | (49.3-88.4) |  |  |  |  |
| Age at end of | 69.6±8.0 | 71.9±7.8 | .17 | 68.6±8.9 | 74.3±9.9 | .003 | .007 | .57 | .22 |
| follow up | (58.0-92.6) | (57.5-88.5) |  | (57.4-92.5) | (57.7-94.7) |  |  |  |  |
| Time followed | 79.5±17.5 | 77.3±23.1 | .66 | 77.6±21.1 | 72.3±30.4 | .86 | .82 | .49 | .71 |
| (months) | (26-92) | (2-92) |  | (6-91) | (0-91) |  |  |  |  |
| Sex (male) | 22 (47.8) | 18 (43.9) | .71 | 37 (74.0) | 34 (69.4) | .61 | .004 | .008 | .02 |
| Obesity | 27 (57.8) | 24 (60.0) | .90 | 20 (40.0) | 23 (46.9) | .49 | .16 | .07 | .22 |
| **Smoking** |  |  | .36 |  |  | .005 | .02 | .12 | .10 |
| Past | 8 (17.4) | 3 (7.3) |  | 7 (14.0) | 11 (22.4) |  |  |  |  |
| Active | 11 (23.9) | 10 (24.4) |  | 22 (44.0) | 7 (14.3) |  |  |  |  |
| Hyperuricemia | 15 (32.6) | 13 (31.7) | .93 | 9 (18.0) | 14 (28.6) | .21 | .35 | .10 | .75 |
| Gout | 1 (2.2) | 1 (2.4) | .93 | 2 (4.0) | 2 (4.1) | >.99 | >.99 | >.99 | >.99 |
| Hypertension | 33 (71.7) | 38 (92.7) | .01 | 42 (84.0) | 40 (81.6) | .76 | .08 | .15 | .12 |
| PVD | 3 (6.5) | 1 (2.4) | .36 | 8 (16.0) | 6 (12.2) | .59 | .13 | .14 | .12 |
| Atrial fibrillation | 10 (21.7) | 10 (24.9) | .77 | 6 (12.0) | 9 (18.4) | .38 | .45 | .20 | .49 |
| Hyperlipidemia | 35 (76.1) | 33 (80.5) | .62 | 45 (90.0) | 46 (93.9) | .48 | .05 | .07 | .05 |
| Diabetes | 19 (41.3) | 24 (58.5) | .11 | 23 (46.0) | 26 (53.1) | .48 | .38 | .64 | .60 |

Abbreviations: CHD, coronary heart disease; DISH, diffuse idiopathic skeletal hyperostosis; PVD, peripheral vascular disease.

Figure S1. Prevalence of DISH at enrolment according to age

There was a statistically significant distribution of DISH across age group (χ2=16.60, p=.018).


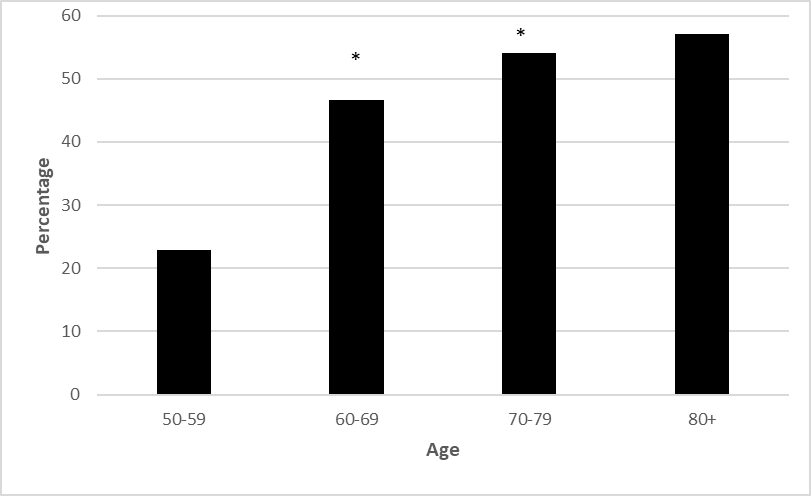


Abbreviations: DISH, diffuse idiopathic skeletal hyperosteosis

* Statistically significantly higher than age 50-59
